# Co-Expression of Lewis y Antigen with Human Epididymis Protein 4 in Ovarian Epithelial Carcinoma

**DOI:** 10.1371/journal.pone.0068994

**Published:** 2013-07-22

**Authors:** Huiyu Zhuang, Jian Gao, Zhenhua Hu, Juanjuan Liu, Dawo Liu, Bei Lin

**Affiliations:** Department of Obstetrics and Gynecology, Shengjing Hospital affiliated to China Medical University, Shenyang, Liaoning Province, China; Florida International University, United States of America

## Abstract

**Objective:**

The main aims of this study were to explore the molecular structural relationship between Human epididymis protein 4 (HE4) and Lewis y antigen by determining their expression patterns and clinical significance in ovarian epithelial carcinoma.

**Methods:**

The structural relationship between HE4 and Lewis y antigen was examined using immunoprecipitation and confocal laser scanning microscopy. HE4 and Lewis y were detected in tissues from malignant (53 cases), borderline (27 cases), benign (15 cases) and normal ovarian tissues (15 cases) using immunohistochemical analysis.

**Results:**

HE4 was present in ovarian cancer, benign tumor tissues, ovarian carcinoma cells, and culture medium, and contained Lewis y antigen. Moreover, expression of Lewis y antigen in HE4 from ovarian cancer was higher than that from benign tumor (*P*<0.05). HE4 possibly exists as two protein isoforms, both containing Lewis y antigen. Our immunohistochemistry data revealed significantly higher positive expression rates of HE4 in malignant ovarian tissues, compared to benign tumor and normal tissue (*P<*0.05), similar to Lewis y antigen levels in ovarian cancer (*P<*0.05). Notably, tissues displaying marked expression of HE4 simultaneously expressed high levels of Lewis y antigen. A linear correlation between the expression patterns of HE4 and Lewis y antigen was evident. Consistently, double-labeling immunofluorescence experiments illustrated co-localization of HE4 and Lewis y antigen within the same area.

**Conclusions:**

HE4 contains Lewis y antigen. Our results further demonstrate a close correlation between the expression levels of the two antigens, which are significantly high in ovarian cancer.

## Introduction

Among the tract malignancies in women, ovarian cancer causes the highest rates of mortality, and diagnosis at the early stages is inadequate. No markers for ovarian cancer with high sensitivity and specificity are currently available to facilitate clinical diagnosis, staging and monitoring for therapeutic interventions. Recent research has disclosed the presence of overexpressed HE4 (human epididymis protein 4) in blood serum of ovarian cancer patients. Expression of human epididymis protein HE4 (WFDC2) in ovarian cancer was initially reported in 1999 [1], and subsequently defined as a biological marker of ovarian cancer in 2003. HE4 was originally discovered in the epithelium of the distal epididymis, and speculated to function as a protease inhibitor in the maturation of sperm [2], [3]. Several researchers further confirmed the utility of HE4 as a specific serum marker [2]–[5]. Additionally, HE4 secreted by ovarian cancer cells was shown to be N-glycosylated [3]. However, several issues, such as the glycosylation status of HE4 in human blood serum, structure, association with the occurrence, development, invasion, migration and resistance of ovarian cancer, and the underlying mechanisms, remain to be established.

Glycosyl antigen, an important component of glycoproteins and glycolipids, is widely expressed in the cell membrane. Changes in the antigen are significantly associated with several biological processes, such as cell canceration, invasion, and migration. In particular, changes in glycosyl type II chain are mainly observed in ovarian cancer. Lewis y antigen, a type of glycosyl antigen, is overexpressed in more than 75% of ovarian epithelial neoplasms to some degree, and high levels are associated with poor prognosis [6]–[8].The tumor markers, CA-125 and MUC-1, also contain Lewis y antigen [9]. Previous experiments by our group showed that Lewis y antigen exists not only in epidermal growth factor receptors, but also other glycoconjugates, such as integrins α5 and β1, as well as CD44 [10]–[12].

In the current study, we investigated the correlation between HE4 and Lewis y antigen with the aid of co-immunoprecipitation and double-label immunofluorescence analyses. Immunohistochemical experiments were simultaneously performed to determine the expression patterns and clinical significance of HE4 and Lewis y antigen in ovarian epithelium carcinoma tissue specimens. Our findings may provide a basis for the effective screening and treatment of ovarian cancer at the early stages.

## Materials and Methods

### Ethics Statement

Samples were fully encoded to protect patient confidentially. The study and its protocols were approved by the Research Ethics committees of Shengjing Hospital Affiliated with China Medical University. IACUC permit number is 2012PS98K. Because all the samples used in the study were discarded, the informed consents were not needed. And the ethics committees approved this.

### Patients and Tissue Samples

Selected paraffin samples (110 in total) and eight fresh samples were obtained from the operations performed from 2000 to 2012 in the Department of Gynecology and Obstetrics of our hospital. All tissue sections were examined by specialists to obtain a final diagnosis. Normal ovarian samples were obtained from tissue excised in cervical cancer operations. Among the benign ovarian tumors, six cases were mucinous cystadenoma, nine cases were serous cystadenoma. There were 27 cases of borderline ovarian tumors (including 13 mucinous and 14 serous cystadenomas). The mean age of these patients was 46.97 years (16–81 years). The age range of the ovarian cancer group was 16 to 73 years; median age was 53 years. The age range of the borderline ovarian tumor group was 22 to 77 years; median age was 36 years. The age ranges of the benign ovarian tumor and normal tissue groups were 22 to 81 years and 37 to 59 years, respectively; median ages were 44 and 50.5 years, respectively. Comparing these groups, there is no statistical significance (P>0.05). Specific histological types and pathological grades are presented in Tables 1 and 2.

**Table 1:** The Expression of HE4 and Lewis y in Different Ovarian Tissues.

Groups	Cases				HE4			Lewis y Antigen					
		–	+	++	+++	Positive Cases	PositiveRate (%)	–	+	++	+++	Positive Cases	Positive Rate (%)
Malignant	53	8	12	16	17	45	84.91*	5	14	18	16	48	90.57#
Borderline	27	10	7	6	4	17	62.96**	11	5	9	2	16	59.26##
Benign	15	12	3	0	0	3	20	10	3	2	0	5	33.33
Normal	15	15	0	0	0	0	0	15	0	0	0	0	0

* Compared with the borderline group. *P = 0.026.*

** Compared with the benign group. *P = 0.008.*

# Compared with the borderline group. *P = 0.001.*

## Compared with benign group. *P = 0.11.*

**Table 2:** Association between HE4 and Lewis y Expression and Pathological Features

Features	Cases	HE4			Lewis y		
		Positive Cases	Positive Rate(%)	*P*	Positive Cases	Positive Rate(%)	*P*
Pathological type							
Mucinous	21	16	76.19	= 0.86	17	80.95	= 0.14
Serous	32	25	78.13		31	96.88	
FIGO stage							
I-II	35	26	74.29	= 0.69	31	88.57	= 0.84
III-IV	18	15	83.33		17	94.44	
Differentiation							
Well	19	11	57.90		13	68.42	
Moderate	19	14	73.68	= 0.5*	17	89.47	= 0.23 #
Poor	15	14	93.33	= 0.02**	15	100.00	= 0.004##
Lymphatic metastasis							
No	44	32	72.73	= 0.55	39	88.64	= 0.57
Yes	9	8	88.89		9	100.00	

* Compared the well- with moderate-differentiation groups.

** Compared the well- with poor-differentiation groups.

# Compared the well- with moderate-differentiation groups.

## Compared the well- with poor-differentiation groups.

Among the fresh specimens, four primary malignant ovarian tumor cases were serous cystadenocarcinoma and four benign cases were serous cystadenoma. According to pathological grading criteria, the ovarian cancer group contained one moderate differentiation and three poor differentiation cases. The group included one stage I and three stage II cases, based on the International Federation of Gynecology and Obstetrics (FIGO) standards.

All cases were primary, and the information was complete. All the patients had received a Gastroscopy or colposcopy to exclude other primaries. Patients were not subjected to chemotherapy prior to the operation. All the patients underwent lymphadenctomy during the operation.

### Co-immunoprecipitation Analysis

SKOV-3 and OVCAR-3 ovarian cancer cell lines were purchased from Cell Culture Collection of ShangHai and propagated in McCoy’s 5A modified medium with 10% fetal bovine serum. Culture medium from SKOV-3 and OVCAR-3 ovarian cancer cells was propagated in medium without fetal bovine serum for 48 h, and malignant and benign ovarian tissues collected. Ice-cold RIPA buffer (1 ml) was added to ovarian cancer cells and tissues separately, followed by incubation at 4°C for 30 min. After centrifugation at 15,000×*g* for 30 min at 4°C, supernatant fractions were collected and treated with anti-HE4 antibody (10 µl) (Santa Cruz, goat polyclonal) for 3 h at 4°C. Protein A/G PLUS-Agarose (20 µl; Santa Cruz) was added, followed by incubation on a rocker platform overnight at 4°C. Beads were collected via centrifugation at 2500×*g* for 5 min and washed. Pellets were resuspended in sodium dodecyl sulfate-polyacrylamide gel electrophoresis (SDS-PAGE) sample buffer and boiled for 5 min. The culture medium was treated with 10 µl HE4 antibody (Santa Cruz, goat polyclonal). All other procedures employed were similar. The negative control contained only 10 µl HE4 antibody (Santa Cruz, goat polyclonal) without protein. Immunoprecipitates were subsequently subjected to 12% SDS gel electrophoresis and analyzed via Western blot using HE4 monoclonal (Abcam, Rabbit) and Lewis y monoclonal (Abcam, Mouse) antibodies. Proteins were visualized using ECL reagent (Amersham ECL Prime detection). Experiments were repeated three times.

### Immunohistochemistry

Histological section of each group of ovarian tissue was 5 µm. Each tissue had two serial sections. Expression patterns of HE4 and Lewis y in ovarian carcinoma tissues were analyzed via immunohistochemical streptavidin-peroxidase staining. Positive and negative immunohistochemistry controls were routinely employed. Normal epididymis tissue served as a positive control for HE4, while gastric cancer tissue was used as the positive control for Lewis y antigen. The negative control was incubated with phosphate-buffered saline instead of primary antibody. The working concentrations of primary antibodies against HE4 and Lewis y used were 1∶40 (Abcam, Rabbit polyclonal to HE4) and 1∶200, respectively. The empirical procedure was performed based on the manufacturer’s instructions.

### Double-Labeling Immunofluorescence Method

The tissue section displayed strong positive expression in immunohistochemistry analysis was selected for the double-labeling immunofluorescence method. The section was simultaneously incubated with primary antibodies against HE4 (1∶100) and Lewis y (1∶100). Negative control sections were incubated with phosphate-buffered saline instead of primary antibody. The working concentrations of fluorescein isothiocyanate and TRITC were 1∶100. Nuclei were counterstained with DAPI. The empirical procedure was performed according to the manufacturer’s instructions.

### Assessment Standard

#### Immunohistochemistry

We consider a positive result if there are buffy granules in the cell membrane and cytoplasm. According to the chromatosis intensity, no pigmentation, light yellow, buffy, and brown are scored 0, 1, 2, and 3, respectively. We choose 5 high-power fields in series from each slice, then score them and take the mean percentage of the chromatosis cells: chromatosis cells that account less than 5% are 0; 5% to 25%, 1; 26% to 50%, 2; 51% to 75%, 3; and greater than 75%, 4. Multiply these 2 numbers; 0 to 2 is considered (−); 3 to 4, (+); 5 to 8, (++); and 9 to 12, (+++). Two observers read the sections to control error. At the same time, we use the NIS-Elements BR 2.10 picture analysis software of the Japanese Nikon Company (Tokyo, Japan) to measure the mean optical density (MOD).

#### Double-Labeling immunofluorescence method

The red fluorescence is HE4 antigen labeled by TRITC, and the green fluorescence is labeled by Lewis y antigen, the blue fluorescence is a nucleus after-stained by DAPI. After taking the pictures, we use the picture analysis software to build up the 3 fluorescence passages; yellow fluorescence illustrates that HE4 and Lewis y antigens are located in the same position.

### Statistical Analysis

SPSS version 17.0 (SPSS Inc, Chicago, IL) software was used for statistical analysis. χ*^2^* analysis, Fisher exact test, variance analysis, and *t*-test were employed. The correlation between HE4 and Lewis y expression in ovarian tumors was examined with the linear regression correlation analysis. *P*<0.05 was considered statistically significant.

## Results

### Co-expression of HE4 and Lewis y Antigen in Ovarian Cancer Tissues, Cells and Culture Medium

**Figure 1:**
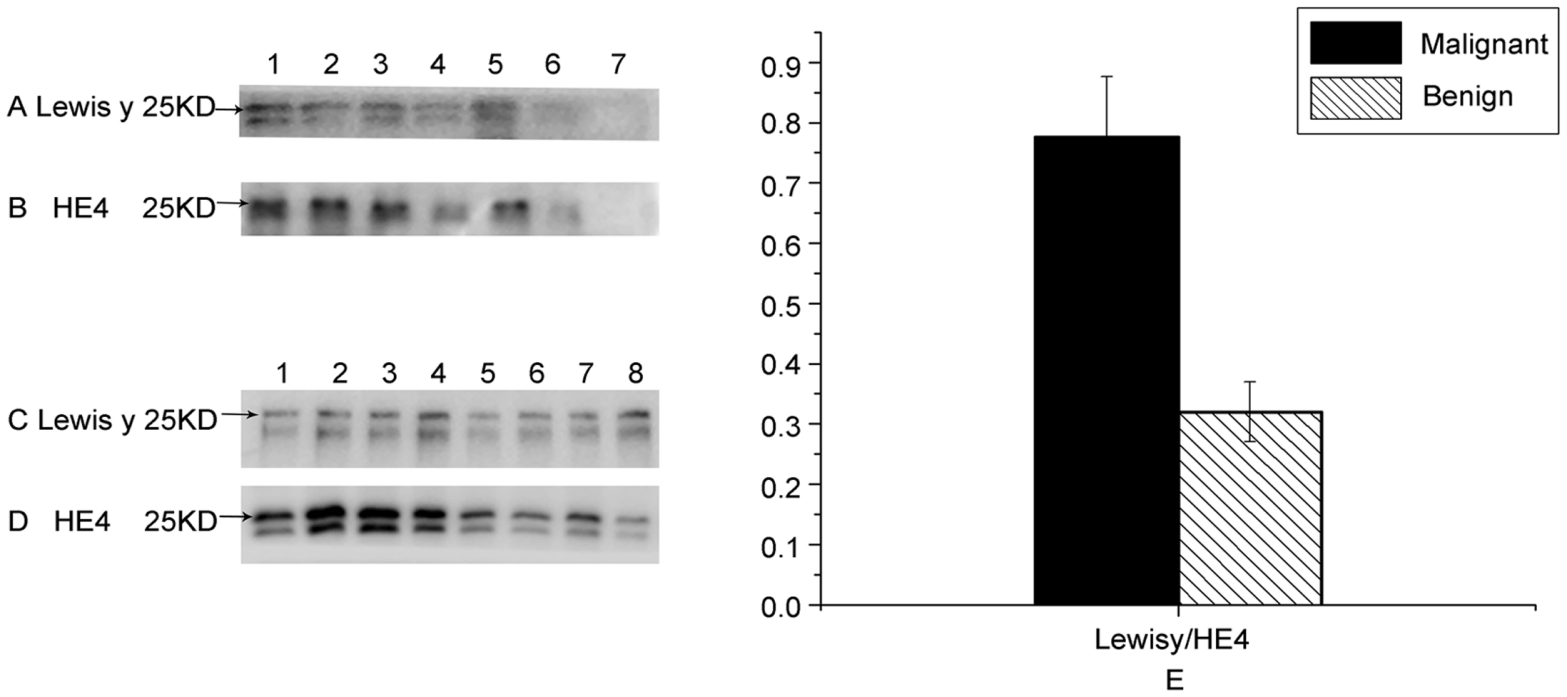
Expression of HE4 and Lewis y in ovarian tissues and cells. In panels A and B, Rows 1 and 2 depict expression of Lewis y antigen and HE4 in ovarian cancer and benign tumor tissues, respectively, Rows 3 and 4 show expression in the ovarian cancer cell line, SKOV3, and culture medium, Rows 5 and 6 in the ovarian cancer cell line, OVCAR3, and culture medium, and Row 7 represents the negative control. In panels C and D, Rows 1 to 4 illustrate expression of Lewis y and HE4 antigen in ovarian cancer tissues, while Rows 5 to 8 show expression in ovarian benign tumor tissues. All the arrows indicate molecular weight (∼ 25 kDa). Quantitative data in panel E are expressed as Lewis y to HE4 in ovarian cancer and benign tumor tissues. The expression of Lewis y antigen in malignant groups was increased by 2.44 times, compared to that in benign groups (*P*<0.05).

Expression patterns of HE4 and Lewis y antigen in HE4 from ovarian cancer tissues, cells and culture medium were examined using the co-immunoprecipitation method. The molecular weight of HE4 was approximately 25 kDa, which included Lewis y (Figure 1). Our data indicate that HE4 possibly exists as two protein isoforms with a molecular weight difference of approximately 3 kDa. Expression levels of the two isoforms in ovarian cancer tissues were equivalent. However, expression was lower in ovarian cancer cells and culture medium. Both HE4 isoforms contained Lewis y, and expression of both HE4 and Lewis y in ovarian cancer groups was higher than that in benign groups (all *P*<0.05). The Lewis y antigen: HE4 expression ratio was employed to calculate the relative amount of Lewis y antigen contained in HE4. Notably, expression of Lewis y antigen in malignant groups was increased by 2.44 times, compared to that in benign groups (*P*<0.05) (Figure 1–E). No HE4 or Lewis y expression was observed in negative controls.

### Expression Patterns of HE4 and Lewis y in Ovarian Tissue Groups

**Figure 2:**
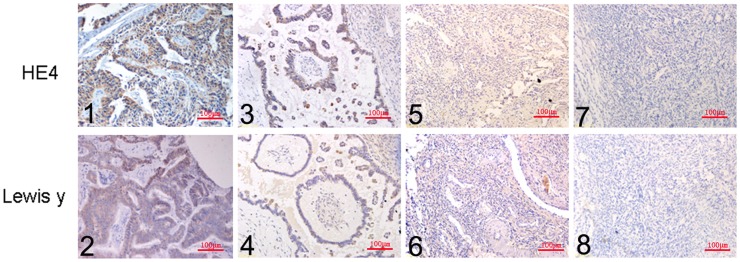
Expression Patterns of HE4 and Lewis y in ovarian tissue groups. Immunohistochemical staining in ovarian malignant tumor (1, 2), borderline tumor (3, 4), benign tumor (5, 6), and normal ovarian tissue (7, 8). HE4 (1, 3, 5, 7) and Lewis y (2, 4, 6, 8; original magnification ×200).

HE4 was mainly localized in the cell membrane and cytoplasm, and detected to a limited extent in the perinuclear region (Figure 2). The positive expression rates in malignant, borderline, benign and normal ovarian tissues of HE4 were 84.91%, 62.96%, 20% and 0%, respectively. Malignant groups displayed the highest positive expression and significantly higher than the rate of the borderline (*P = 0.026*). Expression rates in borderline groups were markedly higher than that in benign groups (*P = 0.008*). No HE4 expression was detected in normal groups.

Lewis y antigen was mainly detected in the cell membrane and cytoplasm (Figure 2). Expression of Lewis y was similar to that of HE4 (generally up to 90.57%), and significantly higher than the rate of the borderline (59.26%, *P = 0.001*). Differences were not significant between the borderline and benign tumor groups (*P = 0.11*). Lewis y was not expressed in the 15 normal ovarian tissue cases (Table 1).

### Correlation of HE4 and Lewis y Antigen Expression with Clinical Features of Ovarian Cancer

In ovarian serous and mucinous cystadenocarcinomas, the positive expression rates of HE4 were 78.13% and 76.19%, respectively, which were similar (*P = 0.86*). HE4 was detected in 83.33% of stage III to IV ovarian cancer cases. The rate of expression was higher than that in stages I to II (74.29%), but this difference did not reach statistical significance (*P = 0.69*). Expression rates of HE4 in the well, moderate, and poor differentiation groups were 57.90%, 73.68% and 93.33%, respectively. The expression rate in the poor differentiation group was thus higher than that in well differentiation group (*P = 0.02*). The positive rate of HE4 in the lymphatic metastasis group (88.89%) was higher than that that of the non-lymphatic metastasis group (72.73%), although this difference was not significant in statistical analysis (*P = 0.55*).

The positive expression rates of Lewis y antigen in ovarian serous and mucinous cystadenocarcinomas were 96.88% and 80.95%, respectively. However, the differences between these expression rates were not significant (*P = 0.14*). The Lewis y antigen was detected in all cases of stage III to IV ovarian cancer at a higher rate, compared to stages I to II (88.57%). The expression rates of Lewis y in the well, moderate, and poor differentiation groups were 68.42%, 89.47%, and 100%, respectively. Antigen levels were up-regulated with decreasing differentiation levels. No significant differences were evident in statistical analysis (*P = 0.57*), despite the higher positive rate of Lewis y antigen presence in the lymphatic metastasis group (100%), compared to that in the non-lymphatic metastasis group (88.64%) (Table 2).

### Relevance of HE4 and Lewis y Expression in Ovarian Cancer

In total, 42 cases simultaneously expressed HE4 and Lewis y positively and two cases displayed simultaneous negative expression among all 53 cases of ovarian cancer (Table 3).Linear regression and correlation analysis revealed that the expression intensities of Lewis y and HE4 show a linear correlation (*r = *0.858, *P*<0.01).

**Table 3:** Relevance of HE4 and Lewis y Expression in Ovarian Cancer.

Lewis y	HE4		Total
	Positive	Negative	
**Positive**	42	6	48
**Negative**	3	2	5
**Total**	45	8	53

**Figure 3:**
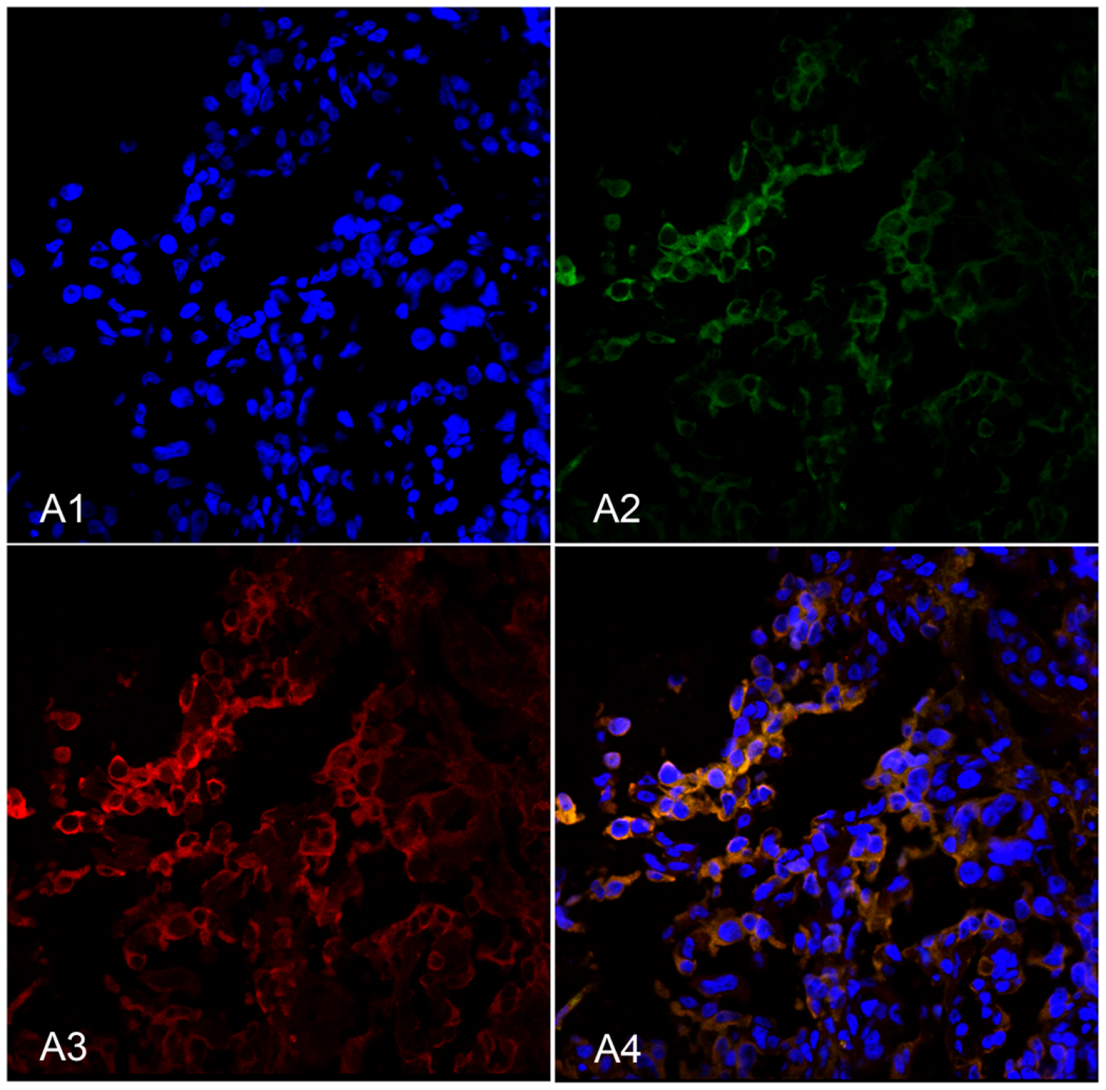
HE4 and Lewis y colocalize in ovarian malignant tumors using double-labeling immunofluorescence. Nucleus (A1), Lewis y (A2), HE4 (A3) and merged image (A4).

In double-labeling immunofluorescence experiments, red fluorescence-labeled HE4 was localized in the cell membrane and cytoplasm, while green fluorescence-labeled Lewis y also appeared in the cell membrane, but was observed to a limited extent in the cytoplasm, and the blue fluorescence was nucleus after-stained by DAPI. Images were obtained, and picture analysis software used to build up three fluorescence passages: yellow fluorescence appeared in the positions where red and green fluorescence overlapped simultaneously. Our findings clearly illustrate that HE4 and Lewis y co-localize at the same positions (Fig. 3).

## Discussion

In 2003, HE4 was detected in the serum of ovarian cancer patients by the group of Hellstrom using ELISA [2]. This novel ovarian tumor protein has been a considerable focus of research over the recent years, and proposed as an effective serologic marker for ovarian cancer diagnosis [4]–[5]. Gene expression profiles indicate that HE4 is one of the most frequently upregulated genes in epithelial ovarian carcinomas. The protein is highly expressed in ovarian epithelium cancer, and conversely, expressed at low levels in benign ovarian tumors. Based on these patterns, HE4 can be clearly utilized as a marker for the early diagnosis of ovarian cancer and assessment of therapeutic effects. Drapkin et al. (2005) reported that culture medium from ovarian carcinoma cells contains a secreted form of HE4 that is N-glycosylated [3] with an approximate molecular weight of 25 kDa. However, several issues, such as whether highly expressed HE4 in serum is in the glycosylated form, its structure and mechanisms of action, are yet to be clarified.

Drapkin and colleagues (2005) reported the presence of HE4 in both the endoplasmic reticulum and Golgi apparatus of ovarian carcinoma cells. It is proposed that HE4 is glycosylated in endoplasmic reticulum and Golgi apparatus of ovarian carcinoma cells and then secreted into culture medium, based on the molecular weights of the protein before and after treatment with the deglycosylation enzyme, N-glycosidase F (PNGase F). Consistent with this theory, we showed that HE4 in culture medium from ovarian carcinoma cells is in the glycosylated form. Moreover, the molecular weight of HE4 in culture medium coincided with those in ovarian cancer tissues and cells, signifying that HE4 is already modified by glycosyl residues. A logical prediction from our studies is that HE4 is glycosylated in the blood of patients with ovarian carcinoma. Our results show for the first time that HE4 is present in ovarian cancer, and benign tumor tissues, ovarian carcinoma cells, and culture medium contain Lewis y antigen. Moreover, expression of the Lewis y antigen component of HE4 from ovarian cancer was higher than that from benign tumor (P<0.05). Our data indicate that the glycoprotein, HE4, possibly exists as two protein isoforms expressed at equivalent levels. However, expression in ovarian cancer cells was lower and the tendency is not equal. This may be attributable to the content and structure of HE4 in tissues. In 2002, analysis of the HE4 gene revealed that at least five putative protein isoforms can be generated via complex alternative splicing [13]. Tokuishi et al. [14] illustrated that expression of the splice variant, HE4–V3, is associated with favorable prognosis in pulmonary adenocarcinoma. Based on the different gene products identified, we proposed the existence of three types of proteins with different molecular weights. However, only two possible proteins containing Lewis y antigen were identified in this study. It is currently unclear whether other isoforms also exist in ovarian cancer or whether these proteins are present in the glycosylated form at the same time. Moreover, we are yet to establish whether the fucosylated form affects the occurrence, development or migration of ovarian cancer.

Among the tissue specimens examined in our study, positive expression rates of HE4 in malignant and boundary ovarian tissues were significantly higher than those in benign tumor, similar to Lewis y antigen levels in ovarian cancer. The positive expression rates in malignant ovarian cancer were markedly higher than those in the borderline, benign tumor and normal tissue groups. The correlation analysis showed that tissues displaying marked expression of HE4 simultaneously express high levels of Lewis y antigen. Overall, a linear correlation (r = 0.858) was observed between HE4 and Lewis y antigen in ovarian cancer. Double-labeling immunofluorescence analyses led to the identification of HE4 and Lewis y antigen within the same locations, further confirming this correlation. Lower degree of ovarian cancer differentiation was associated with higher positive expression rates and intensities of HE4 and Lewis y antigen. An increasing trend of advanced stage cancer was additionally observed.

Evidence obtained from overexpression and knockdown analyses indicates a critical role for HE4 in ovarian cancer cell adhesion, migration and progression, which may be associated with activation of the EGFR-MAPK signaling pathway. Impaired activation of the EGFR and Erk1/2 signaling pathways due to HE4 knockdown could be restored in ovarian carcinoma cells using HE4-containing medium [15]. However, the specific underlying mechanisms remain elusive at present. A previous study by our group [10] showed that overexpression of Lewis y antigen, a component of the structure of EGFR, increases tyrosine phosphorylation of the EGFR receptor and HER2/neu, and promotes signal transduction of the growth factor into cells mainly via PI3K/Akt and Raf/MEK/MAPK signal pathways, leading to increased cell proliferation. In 2004, Klinger [16] showed that an antibody against Lewis y blocks the signal pathway mediated by EGFR and inhibits activation of RAS and phosphorylation of MAPK, in turn, suppressing carcinoma cell proliferation. Further studies demonstrated that fucosylated antigens expressed in tumor cells are involved in several cellular functions and related to malignant behavior, including adhesion, recognition, resistance, and cell signal transduction. Increased fucosylated antigens have been shown to promote invasion and spreading of tumor cells [11], [12], [17], [18]. Lewis y antigen, the oligosaccharide of bifucosylation, is regarded as a tumor-associated marker. Expression of Lewis y antigen is significantly increased in most epithelial neoplasms, including ovarian, pancreatic, prostate, colon, and non-small cell lung cancers [19]. Data from our current study suggest that Lewis y antigen which is an important component of HE4 probably plays crucial roles in the proliferation, apoptosis, invasion, migration and resistance of ovarian cancer via the EGFR-MAPK signaling pathway.

AFP is reported to be highly fucosylated and specific in hepatic cancer serum. Diagnosis of hepatic cancer can be significantly enhanced by the detection of Lens culinaris agglutinin A (AFP-L3) [20]–[21], the only tumor marker of liver cancer currently recognized by the American FDA [22]. In contrast, no changes in AFP have been recorded in benign liver disease. A close relationship between abnormal fucosylation of serum protein and liver cancer occurrence and development is suggested. Benign liver disease can additionally be distinguished from liver cancer with the detection of AFP-L3, which is helpful for early diagnosis and assessment of therapeutic effects. Is HE4 secreted by ovarian cancer patients in the highly glycosylated form? Is there a heteroplasmon of HE4 like AFP-L3? Can Lewis y antigen be effectively utilized as a specific marker for monitoring the disease? These issues remain to be resolved.Our preliminary data indicated that Lewis y antigen is a component of HE4, and both are expressed at high levels in ovarian cancer, which was further confirmed using correlation analysis. Lewis y antigen of HE4 may thus contribute to changes in specific biological behaviors, including adhesion and migration of ovarian carcinoma cells, via the corresponding signal transduction pathways. However, the specific mechanisms underlying the activities of HE4 and Lewis y antigen in ovarian cancer development are currently unclear. Further comprehensive research on the relationship between HE4 and Lewis y should provide feasible theories to explain the occurrence and development of ovarian cancer, and aid in clinical diagnosis and assessment. To date, at least five different HE4 gene types have been distinguished, but the structures and functions of the translated proteins are unknown. In the current study, we identified two possible isoforms of HE4 in ovarian cancer. Elucidation of whether other isoforms additionally exist in the glycosylated form in ovarian cancer and identification of the specific isoforms that play crucial roles in the occurrence, development and migration of ovarian cancer cells should facilitate early diagnosis and improve the therapeutic options for ovarian cancer.
